# Distribution and antibiotic-resistance of different *Staphylococcus* species identified by matrix assisted laser desorption ionization-time of flight mass spectrometry (MALDI-TOF MS) isolated from the oral cavity

**DOI:** 10.1080/20002297.2021.1983322

**Published:** 2021-09-26

**Authors:** Katarzyna Garbacz, Maria Wierzbowska, Ewa Kwapisz, Maja Kosecka-Strojek, Marek Bronk, Morteza Saki, Jacek Międzobrodzki

**Affiliations:** a Department of Oral Microbiology, Medical Faculty, Medical University of Gdansk, Gdansk, Poland; b Department of Microbiology, Faculty of Biochemistry, Biophysics and Biotechnology, Jagiellonian University, Krakow, Poland; c Laboratory of Clinical Microbiology, University Clinical Center, Gdansk, Poland; d Department of Microbiology, Faculty of Medicine, Ahvaz Jundishapur University of Medical Sciences, Ahvaz, Iran; e Student Research Committee, Ahvaz Jundishapur University of Medical Sciences, Ahvaz, Iran

**Keywords:** Coagulase-negative staphylococci, cons, *staphylococcus*, mrsa, methicillin resistance, multidrug resistance, antibiotic

## Abstract

Background: The use of antibiotics in dentistry is associated with the emergence and spread of antibiotic-resistant microorganisms, including commensal staphylococci.

Methods: A total of 367 oral samples were collected, from which staphylococci were isolated and identified by using matrix assisted laser desorption ionization-time of flight mass spectrometry (MALDI-TOF). The antibiotic susceptibility of the isolates was determined and molecular characteristics for methicillin-resistant staphylococci was performed.

Results: A total of 103 coagulase-negative staphylococci (CoNS), among them *S. warneri, S. haemolyticus, S. saprophyticus, S. pasteuri, S. epidermidis, S. hominis, S. xylosus, S. equorum, S. kloosii, S. succinus, S. cohnii*, and *S. simulans*, were confirmed by MALDI-TOF. Resistance to most tested antibiotics was statistically higher in CoNS than in *S. aureus* isolates (*P*-value < 0.05). CoNS isolates showed high resistance to penicillin (*S. saprophyticus* 88.9%), erythromycin (*S. haemolyticus* 84.6%), fusidic acid (*S. saprophyticus* 77.8%), co-trimoxazole (*S. epidermidis* 71.4%), gentamicin (*S. warneri* 63.8%), and tetracycline (*S. saprophyticus* 55.6%). Multidrug resistance was largely observed, especially among *S. haemolyticus* and *S. saprophyticus* species. Methicillin-resistance in *S. haemolyticus* (38.5%), *S. saprophyticus* (22.2%) and *S. aureus* (13.5%) was associated with the presence of the *mec*A gene and SCC*mec* type IV or V.

Conclusion: Coagulase-negative staphylococci, especially *S. haemolyticus* and *S. saprophyticus*, seem to be a reservoir of methicillin resistance and multidrug resistance in the oral cavity.

## Introduction

The oral cavity is a poorly understood ecological niche for staphylococci, which, under specific conditions, may become a source of local or systemic infections. Commensal bacteria, among them staphylococci, gain a growing interest in the context of oral health [1,2].

Staphylococci are responsible for the opportunistic community- and hospital-acquired infections. *Staphylococcus aureus*, one of the coagulase-positive staphylococci (CoPS), constitutes the most common cause of human infections, whereas coagulase-negative staphylococci (CoNS) are believed to be less pathogenic. However, under exposure to the so-called infection-facilitating factors, CoNS may undergo a transition from commensals to pathogens. As a result, they can be involved in a variety of infections with various locations, manifestations and outcomes. CoNS can cause severe infections, such as septicaemia, native and prosthetic valve endocarditis, shunt-associated meningitis, osteomyelitis, and urinary tract infections [3,4]. The infections mentioned above occur primarily in immunocompromised patients and persons with indwelling medical devices [5].

Staphylococci are characterizing by alarmingly increasing rates of antibiotic resistance; this problem belongs to the most important issues related to the management of staphylococcal infections worldwide. In particular, the issue of resistance is important in the case of multidrug-resistant (MDR) and methicillin-resistant (MR) strains. Resistance to methicillin is determined by an extra penicillin-binding protein (PBP2a), encoded by the *mecA* gene, located on a mobile genetic element known as staphylococcal cassette chromosome (SCC*mec*). This cassette can be exchanged between various strains of the same species, or even between various staphylococcal species, and can carry genes encoding resistance to other antibiotics [6]. Methicillin-resistant strains cannot be treated with an anti-staphylococcal penicillin (e.g. oxacillin) and show insusceptibility to all β-lactams, including antibiotics with β -lactamase inhibitors and carbapenems. Furthermore, resistance to methicillin is frequently associated with the resistance to other groups of antibiotics (macrolides, lincosamides, aminoglycosides, fluoroquinolones, and sulfonamides), and requires the use of second-line antibiotics such as vancomycin, daptomycin, or linezolid [7,8].

Recent reports indicate the oral cavity as a significant reservoir for antibiotic-resistant bacteria, especially methicillin-resistant *S. aureus* (MRSA). MRSA may colonize various ecological niches in the mouth, such as the tongue, oral mucosa, periodontal pockets, and denture surfaces [1]. Coagulase-negative staphylococci are known as oral commensal bacteria but the studies reporting the prevalence of staphylococcal species in the oral cavity and their antibiotic resistance are sparse. Consequently, this study aimed to assess the prevalence, antimicrobial-resistance profiles, and molecular characteristics of different *Staphylococcus* species isolated from oral samples.

## Materials and methods

### Isolation of staphylococcal isolates

The oral *Staphylococcus* spp. were isolated from 367 oral microbiological samples analysed consecutively at the Laboratory of Department of Oral Microbiology of the Medical University of Gdansk during routine clinical laboratory procedures, over a period of one and a half years. The analysed staphylococci were not specifically isolated for this research, they were part of the diagnostic laboratory procedure and no humans were involved in the experiments. All samples were streaked onto Columbia blood agar (GrasoBiotech, Starogard Gd., Poland) and differential-selective media mannitol salt agar (bioMérieux, Marcy l’Etoile, France) and were incubated 18–24 h at 37°C. After incubation, colonies with typical staphylococcal morphology (size, shape, or color) were selected to identify by using MALDI-TOF MS according to the manufacturer’s recommendations. The isolates were stored at −80°C in Trypticase Soy Broth (Becton Dickinson, Franklin Lakes, NJ, USA) supplemented with 20% glycerol.

### Identification of staphylococcal isolates by MALDI-TOF MS

The staphylococcal isolates were identified using MALDI-TOF MS (Bruker Daltonics, Germany). Bacteria were prepared for identification by extraction of proteins with ethanol and formic acid, according to the manufacturer’s instructions. One loopful of a fresh culture (20–24 h growth on Brain Heart Infusion Agar at 37°C) was suspended in 150 μl of sterile deionized water, and then 450 μl of pure ethanol was added and the sample was mixed thoroughly by vortexing. After centrifugation, the bacterial pellet was resuspended in 50 μl of 70% aqueous formic acid, then 50 μl of acetonitrile was added to the precipitate, and the sample was thoroughly mixed by vortexing. After centrifugation, 1 μl of the supernatant was collected, applied to a metal plate, and allowed to dry at room temperature. Then, 1 μl of an α-cyano-4- hydroxycinnamic acid matrix solution was applied and the sample was left to dry at room temperature. Calibration was performed using a standard calibration mixture of an *Escherichia coli* extract (Bruker Daltonics) containing RNase and myoglobin proteins. The analysis was repeated three times for each sample. The metal plate with the samples was placed in a MALDI Biotyper chamber for analysis. Automatic measurement of the spectrum and comparative analysis with reference spectra of bacteria were performed using an Ultraflextreme mass spectrometer and MALDI-Biotyper 3.0 software (Bruker Daltonics). The reliability of identification in the MALDI Biotyper system was expressed in points. A log(score) ≥2.0 indicated identification to the species level, and a log(score) ≥1.7 and <2.0 indicated identification to the genus level.

### Antimicrobial susceptibility testing

The antimicrobial susceptibility was performed by the disk diffusion method according to the European Committee on Antimicrobial Susceptibility Testing (EUCAST) [9]. Fifteen antimicrobial agents on Mueller-Hinton agar plates (Becton Dickinson, Franklin Lakes, NJ, USA) were tested: oxacillin, cefoxitin, gentamicin, erythromycin, clindamycin, tetracycline, chloramphenicol, ciprofloxacin, trimethoprim/sulfamethoxazole, fusidic acid, linezolid, rifampicin, tigecycline and vancomycin (Bio-Rad, Marnes la Coquette, France) and penicillin G (Oxoid, Basingstoke, England). The phenotype of resistance to macrolide-lincosamide-streptogramin B was tested and interpreted according to the EUCAST. Vancomycin susceptibility was determined with E-test method (bioMerieux, Marcy-l’Etoile, France). Multidrug resistance (MDR) was defined as a resistance to three or more classes of antibiotics.

### Isolation of staphylococcal DNA

Genomic DNA extraction was performed on each staphylococcal isolate using the Genomic Micro AX Staphylococcus Gravity kit (A&A Biotechnology, Gdynia, Poland) following the manufacturer’s instructions.

### *Methicillin-resistance and staphylococcal cassette chromosome* mec *(SCC*mec*) typing*

Initially, resistance to methicillin was determined using cefoxitin (30 µg) and oxacillin (1 µg) disks, and then confirmed by the detection of PBP2a protein (OXOID ™ PBP2 ‘Latex Agglutination Test Kit, Basingstoke, England). Methicillin-resistance was verified by the detection of the *mec*A and *mec*C genes [10,11]. Methicillin-susceptible *S. aureus* ATCC25923 and methicillin-resistant *S. aureus* ATCC43300 were used as the reference strains. For *mec* positive strains, five major staphylococcal chromosomal cassette *mec* (I–V) was determined as described by Oliveira et al. [12] and by Milheiriço et al. [13]. The SCC*mec* type was determined on the basis of the band pattern profiles obtained.

### Detection of toxin genes

Detection of toxin genes for methicillin-resistant staphylococci was performed. Genes of the enterotoxins (*sea, seb, sec, sed, see*), toxic shock syndrome toxin-1 (*tst*) were detected as described by Becker et al. [14]. Detection of Panton-Valentine leukocidin genes (*lukS*-PV/*lukF*-PV) was performed as described by Lina et al. [15].

### Spa *typing*

The *spa* typing was performed for methicillin-resistant *S. aureus* strains as described previously [16]. The method is based on the sequence analysis of variable region of the protein A (*spa*) gene, resulting in *spa* types, assigned by the Ridom StaphType software version 2.2.1 (http://www.ridom.de/ Ridom GmbH, Wurzburg, Germany) and the Ridom SpaServer database (http://spaserver.ridom.de/). The predicted MLST were assigned based on Ridom SpaServer.

### Statistical analysis

All calculations were performed with Statistica 10 package (StatSoft, Tulsa, OK, USA) with the threshold of statistical significance set at *P-*value ≤ 0.05. The significance of differences in the percentages of antibiotic-resistant CoNS and *S. aureus* isolates was verified with Pearson chi-squared test or Fisher exact test.

## Results

### Distribution of staphylococcal species

One hundred ninety-two *Staphylococcus* spp. isolates belonging to 13 staphylococcal species were identified in this study. The most commonly detected species was *S. aureus* (46.4%). A total of 103 coagulase-negative staphylococci (CoNS), among them *S. warneri* (45.6%), *S. haemolyticus* (12.6%), *S. saprophyticus* (8.7%), *S. pasteuri* (7.8%), *S. epidermidis* (6.8%), *S. hominis* (4.9%), *S. xylosus* (4.9%), *S. equorum* (2.9%), *S. kloosii* (1.9%), *S. succinus* (1.9%), *S. cohnii* (1%), and *S. simulans* (1%), were confirmed by MALDI-TOF MS (Table 1).

**Table 1.** Antibiotic resistance of *Staphylococcus* species isolated from the oral cavity

*Staphylococcus* spp. (n)	No. of antimicrobial resistance (%)													
	FOX	PEN	GEN	ERY	CLI	TET	SXT	FAD	CHL	CIP	TGC	RIF	LIN	MDR
*S. aureus* (89)	12 (13.5)	52 (58.4)	44 (49.4)	19 (21.3)	18 (20.2)	35 (39.3)	3 (3.4)	1 (1.1)	4 (4.5)	1 (1.1)	-	-	-	23 (25.8)
*S. warneri* (47)	3 (6.4)	26 (55.3)	30 (63.8)	9 (19.1)	-	9 (19.2)	3 (6.4)	1 (2.1)	1 (2.1)	-	-	-	-	3 (6.4)
*S. haemolyticus* (13)	5 (38.5)	10 (76.9)	7 (53.8)	11 (84.6)	6 (46.2)	3 (23.1)	5 (38.5)	1 (7.7)	-	3 (6.4)	-	-	-	8 (61.5)
*S. saprophyticus* (9)	2 (22.2)	8 (88.9)	3 (33.3)	5 (55.6)	-	5 (55.6)	1 (11.1)	7 (77.8)	4 (44.4)	-	-	-	-	5 (55.6)
*S. pasteuri* (8)	-	6 (75)	5 (62.5)	5 (62.5)	-	-	1 (12.5)	-	-	-	-	-	-	-
*S. epidermidis* (7)	1 (14.3)	5 (71.4)	1 (14.3)	5 (71.4)	2 (28.6)	2 (28.6)	5 (71.4)	3 (42.9)	-	-	-	-	-	2 (28.6)
*S. hominis* (5)	2/5	3/5	2/5	2/5	1/5	1/5	2/5	1/5	-	-	-	-	-	2/5
*S. xylosus* (5)	-	3/5	2/5	-	-	-	-	-	-	-	-	-	-	-
*S. equorum* (3)	-	2/3	-	1/3	1/3	1/3	-	-	-	-	-	-	-	-
*S. kloosii* (2)	-	1/2	2/2	-	-	1/2	-	1/2	-	-	-	-	-	-
*S. succinus* (2)	1/2	2/2	2/2	1/2	1/2	1/2	-	-	-	-	-	-	-	2/2
*S. cohnii* (1)	-	1/1	-	1/1	-	-	-	-	-	-	-	-	-	-
*S. simulans* (1)	-	1/1	-	-	-	-	-	-	-	-	-	-	-	-
Total (n = 192)	26 (13.5)	120 (62.5)	98 (51)	59 (30.7)	29 (15.1)	58 (30.2)	20 (10.4)	15 (7.8)	9 (4.7)	4 (2.1)	-	-	-	45 (23.4)

CIP – ciprofloxacin, CLI – clindamycin, ERY – erythromycin, FAD – fusidic acid, FOX – cefoxitin, GEN – gentamicin, LIN – linezolid, RIF – rifampicin, SXT – sulfamethoxazole/trimethoprim, TET – tetracycline, TGC – tigecycline, MDR – multidrug resistant

### Antimicrobial resistance

**Figure 1.**
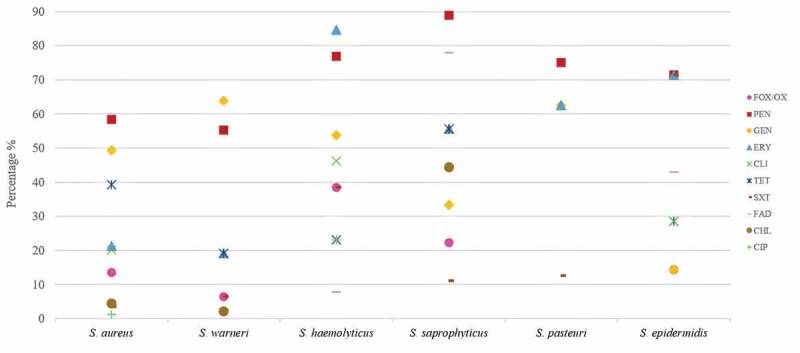
Antibiotic resistance of the six most frequent *Staphylococcus* species isolated from the oral cavity

Overall, the 192 *Staphylococcus* spp. isolates showed resistance to penicillin (62.5%), gentamicin (51%), erythromycin (30.7%), tetracycline (30.2%), cefoxitin/oxacillin (13.5%), clindamycin (15.1%), trimethoprim/sulfamethoxazole (10.4%), fusidic acid (7.8%), chloramphenicol (4.7%), and ciprofloxacin (2.1%). None of the isolates were resistant to linezolid, rifampicin, tigecycline and vancomycin. Resistance to most tested antibiotics was statistically higher in CoNS than in *S. aureus* isolates (*P* < 0.05) (Figure 1). Some CoNS species exhibited especially high resistance to penicillin (*S. saprophyticus* 88.9%), erythromycin (*S. haemolyticus* 84.6%), fusidic acid (*S. saprophyticus* 77.8%), trimethoprim/sulfamethoxazole (*S. epidermidis* 71.4%), gentamicin (*S. warneri* 63.8%), and tetracycline (*S. saprophyticus* 55.6%) (Table 1, Figure 1).

Multidrug resistance was largely observed among *S. haemolyticus* (61.5%), *S. saprophyticus* (55.6%), *S. succinus* (2/2), *S. hominis* (2/5), and *S. aureus* isolates (25.8%) and turned out to be higher in CoNS than in *S. aureus*. MDR CoNS isolates were resistant to 7, 6 and 5 groups of antibiotics (Table 2).

**Table 2.** Antibiotic resistance profiles of methicillin-resistant and multidrug-resistant oral coagulase-negative staphylococci (CoNS)

CoNS species	MR/MDR	Antimicrobial resistance profile (No. of antibiotics)	Methicillin resistance genes	SCC*mec*	MLS phenotype resistance
*S. epidermidis*	MR/MDR	FOX-ERY- CLI-GEN-TET-FAD-SXT (7)	*mecA* (+),*mecC* (-)	SCCmec IV	cMLS_B_
*S. haemolyticus*	MR/MDR	FOX-ERY-GEN-TET-SXT-FAD-CIP (7)	*mecA* (+),*mec* (-)	SCCmec V	MS_B_
*S. haemolyticus*	MR/MDR	FOX-ERY-CLI-GEN-SXT-CIP (6)	*mecA* (+),*mecC* (-)	SCCmec V	iMLS_B_
*S. haemolyticus*	MR/MDR	FOX-ERY-GEN-TET-SXT-CIP (6)	*mecA* (+),*mecC* (-)	SCCmec V	MS_B_
*S. haemolyticus*	MR/MDR	FOX-ERY-GEN-TET (4)	*mecA* (+),*mecC* (-)	SCCmec V	MS_B_
*S. haemolyticus*	MR	FOX	*mecA* (+),*mecC* (-)	SCCmec V	Ery^S^
*S. haemolyticus*	MDR	ERY-CLI-GEN-TET	-	-	cMLS_B_
*S. haemolyticus*	MDR	PEN-ERY-CLI-GEN	-	-	cMLS_B_
*S. haemolyticus*	MDR	ERY-GEN-TET-SXT	-	-	MS_B_
*S. saprophyticus*	MR/MDR	FOX-ERY-GEN-TET-FAD-CHL (6)	*mecA* (+), *mecC* (-)	NT	MS_B_
*S. saprophyticus*	MDR	PEN-ERY-TET-FAD-CHL	-	-	MS_B_
*S. saprophyticus*	MDR	PEN-ERY-TET-FAD-SXT	-	-	MS_B_
*S. saprophyticus*	MR	FOX-FAD	*mecA* (+), *mecC* (-)	NT	Ery^S^
*S. warneri*	MR/MDR	FOX-GEN-TET	*mecA* (+), *mecC* (-)	SCCmec IV	Ery^S^
*S. warneri*	MR/MDR	FOX-ERY-GEN	*mecA* (+), *mecC* (-)	SCCmec IV	MS_B_
*S. warneri*	MR	FOX-GEN	*mecA* (+), *mecC* (-)	NT	Ery^S^
*S. hominis*	MR/MDR	FOX-ERY-CLI-TET-FAD-SXT (5)	*mecA* (+), *mecC* (-)	SCCmec IV	cMLS_B_
*S. hominis*	MR/MDR	FOX-ERY-SXT	*mecA* (+), *mecC* (-)	SCCmec IV	iMLS_B_
*S. succinus*	MR/MDR	FOX-GEN-TET	*mecA* (+), *mecC* (-)	NT	Ery^S^

FOX – cefoxitin, CIP – ciprofloxacin, CLI – clindamycin, ERY – erythromycin, FAD – fusidic acid, GEN – gentamicin, SXT – sulfamethoxazole/trimethoprim, TET – tetracycline, CoNS – coagulase-negative staphylococci, MR – methicillin-resistant, MDR – multidrug resistant, SCC*mec* – staphylococcal cassette chromosome *mec*, MLS – macrolide-lincosamide-streptogramin, NT- non-typeable

Resistance to macrolide-lincosamide-streptogramin was represented by cMLSB (9.7%) and iMLSB phenotypes (1.9%) in CoNS strains, including *S. haemolyticus, S. epidermidis, S. hominis, S. equorum, S. succinus* species. The remaining 29 CoNS isolates (28.2%) resistant to erythromycin represented an MS_B_ resistance profile.

### *Methicillin-resistance and SCC*mec *typing*

Fourteen CoNS isolates were identified as methicillin-resistant (MR), including *S. haemolyticus* (38.5%), *S. saprophyticus* (22.2%), *S. epidermidis* (14.3%), *S. warneri*, (6.4%), *S. hominis* (2/5) and *S. succinus* (1/2) species. All of MR isolates were *mec*A-positive, while none harboured *mec*C. Staphylococcal cassette chromosome *mec* (SCC*mec*) types IV (5/10) and V (5/10) were detected. No isolate represented I, II and III SCC*mec* types. No SCC*mec* type was identified in the case of the four *mec*A-positive CoNS (Table 2).

Twelve of the 89 *S. aureus* isolates were *mec*A-positive and *mec*C-negative. SCC*mec* types IV (66.7%) and V (33.3%) were detected, suggesting a community origin (CA-MRSA). MRSA assigned to nine *spa* types (t012, t091, t156, t189, t437, t888, t5644, t13670 and t18953). MRSA isolates showed _i_MLSB and cMLSB phenotypes of resistance to macrolide-lincosamide-streptogramin (Table 3).

**Table 3.** Characteristics of methicillin-resistant *S. aureus* (MRSA) isolated from the oral cavity

*spa* type	Predicted ST	Methicillin resistance genes	SCC*mec*	Toxin genes	Antimicrobial resistance profile	MLS phenotype resistance
t012	ST-30	*mecA* (+),*mecC* (-)	SCCmec V	*tst, seg, sei sem, sen, seo*	FOX-TET-GEN	Ery^S^
t13670	-	*mecA* (+),*mecC* (-)	SCCmec IV	none	FOX-GEN	Ery^S^
t156	ST-12	*mecA* (+),*mecC* (-)	SCCmec IV	*sec*	FOX-GEN	Ery^S^
t888	-	*mecA* (+),*mecC* (-)	SCCmec V	*sec*	FOX	Ery^S^
t091	ST-7	*mecA* (+),*mecC* (-)	SCCmec IV	none	FOX	Ery^S^
t189	-	*mecA* (+),*mecC* (-)	SCCmec IV	*seg, sei sem, sen, seo*	FOX-TET-GEN	Ery^S^
t437	ST5/ST225	*mecA* (+),*mecC* (-)	SCCmec V	*pvl, seb, sek*	FOX-ERY-CLI	cMLS_B_
t437	ST 398	*mecA* (+),*mecC* (-)	SCCmec V	*pvl, seb, sek*	FOX-TE-GE-SXT	Ery^S^
t5644	-	*mecA* (+),*mecC* (-)	SCCmec IV	*sec*	FOX-GEN	Ery^S^
t18953	-	*mecA* (+),*mecC* (-)	SCCmec IV	*seg, sei sem, sen, seo*	FOX	Ery^S^
t693	-	*mecA* (+),*mecC* (-)	SCCmec IV	*seg, sei sem, sen, seo,eta*	FOX-E-CLI-TET	iMLS_B_
t693	-	*mecA* (+),*mecC* (-)	SCCmec IV	*seg, sei sem, sen, seo*	FOX-E-CLI-TET	iMLS_B_

FOX – cefoxitin, CLI – clindamycin, ERY – erythromycin, GEN – gentamicin, SXT – sulfamethoxazole/trimethoprim, TET – tetracycline, MLS – macrolide-lincosamide-streptogramin, SCC*mec* – staphylococcal cassette chromosome *mec*, MLS – macrolide-lincosamide-streptogramin

### Detection of toxin genes

Genes encoding for the enterotoxin *seb* (2 isolates), enterotoxin *sec* (3 isolates), Panton-Valentine leukocidin (2 isolates), exfoliative toxin A *eta* (1 isolates), and toxic shock syndrome toxin-1 *tst* (1 isolates) were identified in MRSA isolates. Two isolates carrying *lukS*-PV/*lukF*-PV genes represented *spa* type t437 (Table 3).

## Discussion

Staphylococci can be frequently isolated from oral cavities of both healthy and ill persons [17–19]. Recent studies showed that the oral cavity should be considered a potential source of systemic bacterial spread and a reservoir of antimicrobial resistance genes [20–22].

This study demonstrated a relatively high prevalence of diverse staphylococcal species in the oral cavity. Similar to previous studies, we identified *S. aureus* as the most frequent oral isolate [17,23]. Aside from *S. aureus*, we isolated also twelve different species of CoNS. The presence of various CoNS species in the oral cavity was also demonstrated in recent studies conducted in Poland, Japan, and Argentina [23–25]. In a study of healthy persons and patients subjected to kidney transplantation, Majchrzak *et al*. identified thirteen staphylococcal species, with the most frequently isolated CoNS being *S. epidermidis* (40.2%), *S. warneri* (10.3%), and *S. haemolyticus* (9.2%) [24]. Ohara-Nemoto *et al*. reported on nine *Staphylococcus* species; *S. epidermidis* was the most common species isolated from plaque and saliva, followed by *S. hominis, S. warneri, S. intermedius, S. capitis*, and *S. haemolyticus* (12.5–7.1%) [17]. Also, Cuesta et al. [25] identified *S. epidermidis* as the primary CoNS species in the oral cavity of dental patients. The most commonly detected non-*S. aureus* species in our present study were *S. warneri* (45.6%), *S. haemolyticus* (12.6%), and *S. saprophyticus* (8.7%). The discrepancy between our findings and the results of the studies mentioned above may result from differences in patient groups, methods of staphylococci isolation and identification, and geographic region. *S. warneri* was previously reported as a cause of catheter-related bacteriemia, endocarditis, multiple abscesses, and septic arthritis [26,27]. *S. saprophyticus* and *S. haemolyticus* are considered harmful hospital pathogens that cause severe infections with a significant level of bacteraemia [28]. This evidence suggests that the CoNS colonising oral cavity may pose a substantial risk of infection, whether local or systemic one.

While antibiotic resistance of clinical staphylococcal isolates associated with various infections is a well-established fact, little is known about the antibiotic resistance of commensal CoNS present in the oral cavity. In our study, the majority of CoNS (66.1%) and *S. aureus* (51.7%) isolates from the oral cavity were resistant to penicillin. Resistance to most antibiotics was statistically higher in CoNS than in *S. aureus* isolates. Some CoNS species exhibited especially high resistance to tested antibiotics, *S. saprophyticus* being predominantly resistant to penicillin, tetracycline, fusidic acid and chloramphenicol, *S. haemolyticus* to erythromycin and clindamycin, *S. warneri* to gentamycin, *S. epidermidis* to co-trimoxazole. These findings confirm the reports by Cui et al. [28], Arredondo et al. [29], and Szczuka et al. [30], which showed a high level of antibiotic resistance among CoNS species.

Also, the proportion of isolates resistant to multiple antibiotics varied depending on the species. The largest proportions of multidrug-resistant CoNS isolates were found in *S. haemolyticus* (61.5%) and *S. saprophyticus* (55.6%), respectively. Equally high proportions of multidrug-resistant isolates from those species were previously reported by other authors in a clinical setting [3,30]. Our present study adds to those findings, demonstrating that multidrug resistance may also be a common problem in commensal non-healthcare-associated CoNS from the oral cavity and does not necessarily result from antibiotic pressure.

Methicillin-resistant staphylococci constitute a major challenge in the treatment of both nosocomial and community-acquired infections. Methicillin resistance is determined by an extra penicillin-binding protein (PBP2a), encoded by the *mecA* gene. In the present study, the *mec*A gene was found in 38.5%, 22.2%, 13.5% *S. haemolyticus, S. saprophyticus* and *S. aureus* isolates, respectively. The proportions of isolates carrying this gene were nearly thrice as high as reported by Smith *et al*. [19] but still lower than the rates documented in staphylococcal infections (40–60%) [8,28]. In previous studies, *mec*A-carriage was frequently demonstrated in CoNS belonging to S. *haemolyticus, S. epidermidis*, and *S. hominis* species, which is consistent with our findings [5,30,31]. *mec*A gene carriage rate in *S. saprophyticus* (22.3%) was higher than in clinical isolates reported by Cui et al. [28]. The presence of the *mecA* gene was not observed in some staphylococcal species, such as *S. xylosus, S. pasteuri, S. simulans*, and *S. cohnii*, which is consistent with the previous results [32]. The MDR phenotype was widespread among methicillin-resistant CoNS strains, such as *S. haemolyticus S. saprophyticus*, and *S. epidermidis*. These results imply that CoNS may constitute a potential reservoir for methicillin resistance and play a role in the inter-species transfer of resistance genes.

SCC*mec* is a mobile genetic element consisting of two components, the *mec* gene complex and *the ccr* (cassette chromosome recombinase) gene complex. The combination of the genes confers various SCC*mec* types. SCC*mec* types I, II and III are predominant in hospital-acquired isolates (HA-MRSA), whereas SCC*mec* types IV and V are mainly associated with community-acquired isolates (HA-MRSA). SCC*mec* types IV and V are smaller than SCC*mec* types I, II and III, which facilitates their mobility and spread [6]. While SCC*mec* types I, II and III were not identified in the present study, *S. aureus* and CoNS isolates were shown to harbour SCC*mec* type IV or V. SCCmec type V was preferentially associated with *S. haemolyticus*, similar to results demonstrated by Szczuka et al. [30]. No SCC*mec* type was identified in the case of the four *mec*A-positive CoNS. These findings are consistent with the results of previous studies in which non-typeable *ccr* genes were shown to be associated with the heterogeneity of SCC*mec* elements in methicillin-resistant CoNS strains [31,33].

This study demonstrated a relatively high diversity of *spa* types in detected oral MRSA isolates. Particular attention should be given to two isolates of the *spa* type t437 having *lukS*-PV/*lukF*-PV genes. Panton-Valentine leukocidin (PVL) genes is considered as a stable genetic marker for CA-MRSA strains that can carry SCCmec type IV or V. Above half (66.6%) of *S. aureus* isolates in this study were assigned to SCC*mec* IV, whereas strains represented *spa* t437 harbored SCC*mec* V. Similar strains were isolated in Germany and in Taiwan [34,35], but recent report from Poland described predominance of t437 SCC*mec*-IV-*pvl*-positive strains in specimens from diabetic patients [36].

In conclusion, this study showed that the oral cavity is colonised by both *S. aureus* and a broad spectrum of diverse CoNS species. The level of antimicrobial resistance was higher in CoNS than in *S. aureus* isolates. CoNS isolates, especially *S. haemolyticus* and *S. saprophyticus*, often displayed MDR, with high rates of resistance to methicillin, erythromycin, gentamicin, tetracycline, co-trimoxazole and fusidic acid. In both *S. aureus* and CoNS, methicillin-resistance was associated with the presence of the *mec*A gene and SCC*mec* types IV or V. The MRSA isolates identified as *spa* type t437 carried Panton-Valentine leucocidin genes (*lukS*-PV/*lukF*-PV). Although *S. aureus* strains appear to be more prevalent in the oral cavity, these are coagulase-negative staphylococci, especially *S. haemolyticus* and *S. saprophyticus*, which seem to be a reservoir of methicillin resistance and multidrug resistance. Our findings warrant monitoring for both colonisation and resistance rates of staphylococci in the oral cavity.
